# Effect of Bicalutamide Combined with Docetaxel on Serum PSA and VEGF Levels in Patients with Advanced Prostate Carcinoma

**DOI:** 10.1155/2022/4506350

**Published:** 2022-08-17

**Authors:** Zhaoxin Guo, Xiaolin Hu, Renguang Lv, Yongzhen Zhang, Liwei Meng, Zhaoxu Liu, Lei Yan

**Affiliations:** ^1^Department of Urology, Qilu Hospital of Shandong University, Jinan, 250012 Shandong, China; ^2^Department of Endocrinology, Jinan Central Hospital, Jinan, 250033 Shandong, China; ^3^Department of Urology, Jinan Seventh People's Hospital, Jinan, 251400 Shandong, China

## Abstract

**Objective:**

To investigate the effect of bicalutamide combined with docetaxel on the levels of prostate-specific antigen (PSA) in serum and vascular endothelial growth factor (VEGF) in patients with advanced prostate carcinoma (PCa).

**Methods:**

The clinical data of 103 patients with advanced PCa at our hospital between Feb. 2020 and Feb. 2021 were retrospectively analyzed, the 90 of whom screened by inclusion and exclusion criteria were finally chosen as research objects. They were divided into a control group and an experimental group according to the order of admission, with 45 cases in each group. The control group was treated with conventional treatment, while the experimental group underwent the combination of bicalutamide and docetaxel, and the clinical indices of the two groups were compared.

**Results:**

After treatment, the serum indices in the experimental group were remarkably lower than those in the control group (*P* < 0.001), with remarkably lower incidence of toxic and side effects (*P* < 0.05) and higher Expanded Prostate Cancer Index Composite (EPIC) scores (*P* < 0.001) in the experimental group than in the control group.

**Conclusion:**

The implementation of bicalutamide combined with docetaxel in patients with advanced PCa is effective in reducing the inflammatory expression and improving quality of life and has a higher safety profile. Compared with conventional treatment, this method is of high application value, and further studies will help establish a better solution for such patients.

## 1. Introduction

As a common disease in urology, prostate carcinoma (PCa) is the epithelial malignant tumor occurring in the prostate [1]. According to statistics, there were nearly 1.29 million new cases of PCa in 2018, accounting for 6.98% of all tumors, and the number of death cases was as high as 35,4800 [2]. Relevant literature has suggested that PCa is the second most common malignant tumor and the fifth most common mortality among men worldwide [3]. Castellucci et al. [4] have pointed out that there are distinct ethnic regional differences in the incidence and mortality of PCa which ranks as the leading male malignancy in Europe, New Zealand, the Americas, Australia, and most of sub-Saharan Africa, with a disease incidence of 70-85 per 100,000. It is a major contributor to cancer deaths in men in 46 countries, particularly in the Caribbean and sub-Saharan Africa [5]. It is reported that the incidence of PCa in China is eminently lower than that in Western countries but is increasing with changing diets, an aging population, and the development of early screening [6]. Xu et al. [7] have noted that approximately 1.09 million men are diagnosed with PCa and 290,000 die from it each year. It is estimated that the number of new cases of PCa will increase to 1.65 million and deaths will increase to 495,000 by 2030 due to global population growth and aging [8]. Compared to western countries such as the US, China has a lower incidence rate but accounts for 4.99% of deaths from PCa worldwide [9]. PCa can lead to urination disorders and induce systemic damage, such as renal failure and digestive failure, and can also metastasize to other parts of the body through blood and lymph nodes, seriously threatening the health of patients. The cause of this tumor is currently unknown and is presumed to be related to age, genetics, diet, and environmental factors. Therefore, it has become the focus of current medical research to improve the detection rate, reduce the mortality rate, and find reasonable treatments of PCa.

Surgery, radiotherapy, chemotherapy, and androgen deprivation therapy (ADT) are the main clinical treatments for PCa [10]. Surgery is the standard protocol for PCa owing to its ability to rapidly reduce androgen in the patients' body, but surgical castration alone has a high recurrence rate and poor efficacy. In addition, PCa patients are usually diagnosed at an advanced stage and the body functions of the elderly decline, for which reason most of them are intolerant and difficult to undergo surgical treatment, and therefore, palliative therapies based on endocrine treatment are mainly adopted in clinic [11]. Although conventional treatment can obviously improve the clinical symptoms of patients, the therapeutic effect does not reach the expectation and barely meet the clinical demand. Bicalutamide is an antihormone drug with strong specificity that can effectively reduce androgen in the body, and docetaxel enhances the polymerization and inhibits the depolymerization of tubulin and disrupts mitosis of tumor cells [12]; the combined effect of the two is remarkable. Meanwhile, a study has shown that prostate-specific antigen (PSA) can be used to diagnose PCa and determine the effect and prognosis of endocrine treatment, which is a common clinical detection index [13]. Vascular endothelial growth factor (VEGF) can promote the angiogenesis of tumors, which is also an important index factor to judge the prognosis of PCa [14]. Thus, in order to further investigate the effect of bicalutamide combined with docetaxel on serum PSA and VEGF levels in patients with advanced PCa, this paper adopts this scheme and carries out a combined clinical intervention for the research objects, aiming to provide more evidence-based clinical proof for such patients.

## 2. Materials and Methods

### 2.1. General Data

The clinical data of 103 patients with advanced PCa at our hospital between Feb. 2020 and Feb. 2021 were retrospectively analyzed. Among them, 90 cases met the inclusion criteria and 13 cases were excluded. They were divided into a control group and an experimental group according to the order of admission, with 45 cases in each group. The study was in accordance with the Declaration of Helsinki [15].

### 2.2. Recruitment of Research Objects

Inclusion criteria. ① The patients met the diagnostic criteria of PCa in the Diagnosis and Treatment of Prostate Cancer [16] and confirmed by imaging and pathological diagnosis and were identified as advanced PCa by the TNM staging method, with clinical symptoms such as slow urine stream, urinary incontinence, and hematuria; ② the patients had an expected survival period of >1 year and no distant metastasis; ③ the patients were able to better cooperate with the study, related inspection, and follow-up with independent consciousness; ④ the patients had been diagnosed with PCa breaking through the capsule 30 d before inclusion; and ⑤ the patients were treated according to the scheme given by the hospital and had the corresponding treatment indications during the whole process of treatment.

Exclusion criteria. ① Patients who had received chemotherapy or other treatment regimens before; ② patients with psychiatric abnormalities or cognitive impairments that affected treatment and communication; ③ patients with contraindications to treatment or drug allergies; ④ patients with severe cardiac, cerebral, hepatic, or renal insufficiency; ⑤ patients with other malignant tumors; and ⑥ patients with pituitary or adrenal cortical dysfunction.

### 2.3. Methods

#### 2.3.1. Control Group

Patients in the control group were given abiraterone acetate tablets (manufacture: Jiangxi Shanxiang Pharmaceutical Co., Ltd.; NMPA Approval No. H20193276; specification: 0.25 g∗120 tablets/box), once a day, 1000 mg. On this basis, prednisone acetate tablets (manufacture: Xianju Pharmaceutical Co., Ltd.; NMPA Approval No. H33021207; specification: 5 mg∗100 tablets) were introduced, 5 mg-10 mg each time and 10 mg-60 mg per day orally.

#### 2.3.2. Experimental Group

Patients in the experimental group received bicalutamide capsules (manufacture: Shanxi Zhendong Pharmaceutical Co., Ltd.; NMPA Approval No. H20060983; specification: 50 mg∗30 s) with 50 mg each time, once a day. On this basis, an intravenous drip of docetaxel (manufacture: Beijing Eastern Union Biopharmaceuticals, Ltd.; NMPA Approval No. H20050879; specification: 0.5 ml: 20 mg) was administrated. Based on the calculated amount of medication for the patients, the required dose was inhaled with a syringe and diluted into 5% glucose injection or 0.9% sodium chloride injection, gently shaken, and mixed well to a final concentration of ≤0.74 mg/ml. The docetaxel dosage was 70 mg/m^2^-75mg/m^2^, with an intravenous drip of 1 h, once every 3 weeks.

#### 2.3.3. Period of Treatment

Patients in both groups were reviewed once every 30 d. When the PSA index was <0.2 ng/ml, the medication could be stopped, and when the PSA index was >4 ng/ml, the medication needed to be continued, for 12 months.

### 2.4. Observation Indices

After treatment, 5 ml of fasting venous blood was collected from both groups, which was centrifuged at 3000 r/min for 10 min by a centrifuge (model: TD4ZB; manufacturer: Changsha Xiangrui Centrifuge Co., Ltd.), with the upper layer of serum preserved. Serum PSA indices were detected by the electrochemiluminescence method, with VEGF indices detected by enzyme-linked immunosorbent assay. All operations were carried out strictly according to the instructions of the kit (purchased from Wuhan Saipei Biotechnology Co., Ltd.).

The incidence of toxic and side effects was compared between both groups, including gastrointestinal reactions, abnormal liver function, sexual dysfunction, and sensory abnormalities.

The quality of life was assessed by the Expanded Prostate Cancer Index Composite (EPIC) [17], whose evaluation items were sexual function (observing whether the sexual initiative, erectile function, and psychophysiological responsiveness to sex were normal), urinary function (observing the disorders of the ureter, kidneys, urethra, and bladder function), intestinal function (observing the disorders of the intestinal absorption, digestion, and secretion), and hormonal function (observing the levels of the hormone, androgen, and adrenal cortical hormone). The higher the function score, the better the quality of life; the higher the symptom score, the more obvious the symptoms; total score = function score − symptom score, followed by linear conversion, with scores ranging from 0 to 100 and higher scores representing better quality of life.

### 2.5. Statistical Disposal

The data of the study were statistically analyzed by SPSS21.0. The count data were tested with the *x*^2^ test and expressed by ((*n*%)), with measurement data by the *t* test and ($\bar{x}\pm s$). *P* < 0.05 suggested a statistically remarkable difference.

## 3. Results

### 3.1. Comparison of Baseline Data

No remarkable differences were found in the age, BMI, mean disease duration, clinical stages, number of comorbidities, education levels, career, religious beliefs, family income, smoking, drinking, and residence between both groups (*P* > 0.05), see Table 1.

### 3.2. Comparison of Serum Indices after Treatment

After treatment, the serum indices in the experimental group were remarkably lower than those in the control group (*P* < 0.001), see Table 2.

### 3.3. Incidence of Toxic and Side Effects

The incidence of toxic and side effects in the experimental group was remarkably lower than that in the control group (*P* < 0.05), see Table 3.

### 3.4. Comparison of EPIC Scores after Treatment

After treatment, the EPIC scores in the experimental group were remarkably higher than those in the control group (*P* < 0.001), see Table 4.

## 4. Discussion

Significant racial and geographic differences in the incidence of PCa have been reported, and in the US, the incidence of PCa has surpassed lung cancer as the number one tumor endangering men's health [18]. The incidence of PCa in Asia is much lower than that in Europe and the US, but it has been on the rise in recent years and is growing more rapidly than in the latter. Patients with advanced PCa have a poor prognosis with short survival time, and most of them are accompanied by bone metastasis symptoms. Therefore, clinical treatment is aimed at controlling disease progression and improving the survival time and quality of patients as well as their quality of life. Studies have shown that PCa patients lean more on androgen, whose bond with receptors can stimulate the prostate cancer cells to divide and proliferate [19]. Meanwhile, Li et al. [20] have found that the blocking of sources of dihydrotestosterone, of which about 89% originates from the testis and 11% from the adrenal gland, can effectively inhibit the growth of prostate cancer cells. Bicalutamide is a competitive inhibitor of male receptors, which inhibits androgen receptors and induces apoptosis in prostate cancer cells, thus effectively inhibiting their function and growth. Docetaxel is a cytotoxic antitumor drug that achieves the purpose of antitumor by disrupting the equilibrium state of microtubule and tubulin dimer [21]. PSA, a glycoprotein secreted by tissues with prostate cancer, is an important serum index for the evaluation and screening of PCa and is closely related to its occurrence and progression. VEGF is a multifunctional cell growth factor that can promote neovascularization and plays an important role in the formation and maintenance of neovascularization in PCa [22]. The results of this study showed that all serum indices were remarkably lower in the experimental group than those in the control group after treatment (*P* < 0.001), indicating that the continuous implementation of this treatment scheme significantly reduced and maintained serum PSA and VEGF levels. The reasons were analyzed as follows. As an antiandrogen drug, bicalutamide can bind to the androgen receptor in the body with high intensity, thus reducing the growth-promoting effect of testosterone and dihydrotestosterone on cancer cells, maximally controlling the metastasis of prostate cancer cells and consolidating the therapeutic effect. In addition, this treatment can effectively reduce testosterone. Docetaxel, an antitumor drug of taxane compounds, can destroy the mitosis of tumor cells by enhancing tubulin polymerization and inhibiting tubulin depolymerization, thereby inhibiting the division and proliferation of tumor cells, and also reducing the serum PSA and VEGF levels of patients. The combination of the two greatly reduces the serum PSA and VEGF levels of advanced PCa patients, which is positive for the improvement of the disease. Meanwhile, Yang et al. [23] have found that the application of intermittent endocrine treatment in elderly patients with advanced PCa is effective in reducing serum PSA and VEGF levels, regulating immune function, and promoting patients' quality of life, which is consistent with the results of this study.

The incidence of toxic and side effects is an important evaluation index of treatment safety. Gastrointestinal reactions, abnormal liver function, sexual dysfunction, and sensory abnormalities are common adverse reactions in PCa patients, which seriously affect their quality of life. In this study, the overall incidence of toxic and side effects in the experimental group was remarkably lower than that in the control group (*P* < 0.05), indicating that bicalutamide combined with docetaxel is safer than conventional treatment. Moreover, it has been shown that intermittent endocrine treatment for advanced PCa in the elderly can arrest the progression of PCa to the androgen-independent phase, significantly improve the prognosis of patients, and improve their quality of life [24]. The EPIC scale, which is suitable for PCa patients at different stages of development and treatments (mainly surgery, chemotherapy, radiotherapy, and hormonal therapy), is widely used in various countries and regions. The scale is short, is easy to use, and can help clinicians to comprehensively, accurately, rapidly, and specifically assess the quality of life of PCa patients with good reliability and validity, serving as a powerful tool to synthetically assess the quality of health and life related to PCa. Hashimoto et al. [25] have found that the quality of life of most patients with localized PCa within 1 year of treatment tended to decline, and the effect of different treatments on quality of life varied considerably. The results of this study have shown that the scores of sexual function, urinary function, intestinal function, and hormonal function in the experimental group were higher than those in the control group, which fully explains that the combination of bicalutamide and docetaxel is effective in improving the quality of life of patients and is beneficial to their prognosis. Inadequacy of this study: first of all, the selected cases were patients in local hospitals and the source of cases was single; secondly, the study was limited by the observation time, which did not include a sufficient sample size, resulting in bias in the study results; finally, there was a lack of long-term follow-up observation on the intervention effect of patients. Therefore, multicenter clinical research should be conducted to further explore the therapeutic characteristics of bicalutamide combined with docetaxel and assessment tools for bladder function should be added, so as to better exploit the therapeutic advantages of drug combination and benefit more PCa patients.

In summary, bicalutamide combined with docetaxel is a reliable regimen for the treatment of advanced PCa, and the application of this regimen can benefit the patients by effectively reducing serological indexes and improving quality of life. Therefore, it is recommended for the clinical treatment of PCa.

## Figures and Tables

**Table 1:** Comparison of baseline data.

Items	Experimental group (*n* = 45)	Control group (*n* = 45)	*x* ^2^/*t*	*P*
Age ($\bar{x}\pm s$, years)	65.44 ± 6.18	66.42 ± 6.46	0.735	0.464
BMI (kg/m^2^)	19.98 ± 0.53	20.13 ± 0.65	1.199	0.234
Mean disease duration (years)	2.84 ± 1.00	2.80 ± 0.99	0.191	0.849
Clinical stages			0.049	0.824
Stage C	30 (66.67%)	29 (64.44%)		
Stage D	15 (33.33%)	16 (35.56%)		
Number of comorbidities (types)	2.47 ± 1.52	2.33 ± 1.51	0.438	0.662
Education levels			0.048	0.827
High school and above	29 (62.22%)	28 (64.44%)		
Middle school and below	16 (37.78%)	17 (35.56%)		
Career				
Civil servants	9 (20.00%)	10 (22.22%)	0.067	0.796
Workers	8 (17.78%)	9 (20.00%)	0.073	0.788
Farmers	6 (13.33%)	7 (15.56%)	0.089	0.764
Individual business owners	7 (15.56%)	5 (11.11%)	0.385	0.535
Other	15 (33.33%)	14 (31.11%)	0.051	0.822
Religious beliefs			0.062	0.803
Yes	10 (22.22%)	11 (24.44%)		
No	35 (77.78%)	34 (75.56%)		
Family income			0.045	0.832
(≥3000 yuan/man·month)	21 (46.67%)	20 (44.44%)		
(<3000yuan/man·month)	24 (53.33%)	25 (55.56%)		
Smoking			0.216	0.642
Yes	33 (73.33%)	31 (68.89%)		
No	12 (26.67%)	14 (31.11%)		
Drinking			0.073	0.788
Yes	36 (80.00%)	37 (82.22%)		
No	9 (20.00%)	8 (17.78%)		
Residence			0.178	0.673
Urban areas	21 (46.67%)	23 (51.11%)		
Rural areas	24 (53.33%)	22 (48.89%)		

**Table 2:** Comparison of serum indices after treatment ($\bar{x}\pm s$).

Groups	*n*	PSA (ng/ml)	VEGF (pg/ml)
Experimental group	45	5.21 ± 0.55	84.05 ± 6.00
Control group	45	9.36 ± 1.47	94.52 ± 13.35
*t*		17.737	4.799
*P*		<0.001	<0.001

**Table 3:** Incidence of toxic and side effects (*n*(%)).

Groups	*n*	Gastrointestinal reactions	Abnormal liver function	Sexual dysfunction	Sensory abnormalities	Total incidence
Experimental group	45	2 (4.44%)	1 (2.22%)	1 (2.22%)	2 (4.44%)	6 (13.33%)
Control group	45	4 (8.89%)	3 (6.67%)	3 (6.67%)	4 (8.89%)	14 (31.11%)
*x* ^2^						4.114
*P*						<0.05

**Table 4:** Comparison of EPIC scores after treatment ($\bar{x}\pm s$).

Groups	*n*	Sexual function	Urinary function	Intestinal function	Hormonal function
Experimental group	45	85.76 ± 2.19	41.63 ± 2.78	87.93 ± 2.56	77.95 ± 4.49
Control group	45	78.99 ± 2.49	31.84 ± 1.74	79.67 ± 3.49	67.39 ± 4.44
*t*		13.695	20.025	12.802	11.218
*P*		<0.001	<0.001	<0.001	<0.001

## Data Availability

Data to support the findings of this study is available on reasonable request from the corresponding author.
